# Harmonization of Real-World Data for Vaccine-Preventable Infectious Diseases: Integration of SARS-CoV-2 Diagnostic and Serologic Data From Multiple Sources

**DOI:** 10.1093/ofid/ofag454

**Published:** 2026-07-23

**Authors:** Yonah C Ziemba, Suhyeon Yoon, Harvey W Kaufman, William A Meyer, Laura Gillim, Nkemakonam Okoye, Cheryl B Schleicher, Shahidul Islam, Cristina P Sison, Ligia A Pinto, Lynne Penberthy, James M Crawford

**Affiliations:** Northwell Health, Department of Pathology and Laboratory Medicine, New Hyde Park, New York, USA; Integrated Data Sciences Section, Research Technologies Branch, National Institute of Allergy and Infectious Diseases, National Institutes of Health, Bethesda, Maryland, USA; Department of Pediatrics, Harvard Medical School, Boston, Massachusetts, USA; Quest Diagnostics, Secaucus, New Jersey, USA; Labcorp, Burlington, North Carolina, USA; Northwell Health, Department of Pathology and Laboratory Medicine, New Hyde Park, New York, USA; Northwell Health, Department of Pathology and Laboratory Medicine, New Hyde Park, New York, USA; Northwell Health, Feinstein Institutes for Medical Research, Manhasset, New York, USA; Northwell Health, Biostatistics Unit, Office of Academic Affairs, New Hyde Park, New York, USA; Northwell Health, Feinstein Institutes for Medical Research, Manhasset, New York, USA; Northwell Health, Biostatistics Unit, Office of Academic Affairs, New Hyde Park, New York, USA; Frederick National Laboratory for Cancer Research, Frederick, Maryland, USA; Data Axle, Inc., Dallas, Texas, USA; Northwell Health, Department of Pathology and Laboratory Medicine, New Hyde Park, New York, USA; Northwell Health, Feinstein Institutes for Medical Research, Manhasset, New York, USA

**Keywords:** cancer, LOINC, SARDs, standardization, transplantation

## Abstract

**Background:**

The COVID-19 Real World Data infrastructure (CRWDi) was established to study the impact of coronavirus disease 2019 (COVID-19) on patients with immunocompromising conditions. A key challenge was harmonizing severe acute respiratory syndrome coronavirus 2 (SARS-CoV-2) laboratory test results.

**Methods:**

There were 27 different test names for SARS-CoV-2 nucleic acid amplification tests (NAATs), and there were 34 and 26 for SARS-CoV-2 qualitative and semiquantitative serologic tests, respectively. We validated a strategy to eliminate the multiplicity of test names and results, and harmonized SARS-CoV-2 semiquantitative serology test results by target antigen and antibody using published conversion factors to report Binding Arbitrary Units (BAU) on a uniform scale.

**Results:**

For 5 200 000 patients, the numbers of unique SARS-CoV-2 test events were 4 865 431 NAAT, 3 092 198 qualitative, and 834 487 semiquantitative serology tests, of which 378 522 were antispike (anti-S) semiquantitative serology. In achieving harmonization of semiquantitative serologic results, semi-log cumulative ordinal plots demonstrated that test results from patients with transplantation exhibited a significantly higher proportion of negative-and-submaximal SARS-CoV-2 anti-S BAU values when compared with patients with cancer or systemic autoimmune or rheumatic diseases (SARDs) or with a general nonimmunocompromised population.

**Conclusions:**

Complex and heterogeneous test name and result conventions can represent a significant barrier to understanding the clinical relevance of real-world laboratory data. The generalizable methodology described herein permitted successful harmonization of SARS-CoV-2 diagnostic and serologic test data from multiple laboratory sources with administrative claims data, vital status data, and structured data on cancer diagnostics and treatment. This methodology supports exploration of the clinical utility of semiquantitative serologic data, especially in patients with immunocompromising conditions.

Infectious diseases are part of our species’ existence. Regardless of whether pathogens are previously identified or are novel, tracking their impact on human populations is essential for management of public health and for informing medical practice [1]. Established national and global public surveillance networks can adapt quickly to the detection and monitoring of emergent novel pathogens [2]. However, the recent coronavirus disease 2019 (COVID-19) pandemic demonstrated the challenges of having an extant data infrastructure to develop real-world evidence to inform medical practice. Fortunately, productive multi-institutional consortia were quickly established that addressed key data needs for understanding and managing the COVID-19 pandemic [3, 4], including tracking the effectiveness of vaccination for administration to the public [5, 6].

However, important questions persisted through the pandemic, including the impact of COVID-19 on immunocompromised patients [7, 8] and the utility of severe acute respiratory syndrome coronavirus 2 (SARS-CoV-2) serologic testing in assessing the host immune response following either natural infection or vaccination [9, 10]. In the first instance, US-based studies have provided strong evidence regarding the risk of immunocompromised patients for severe illness and postacute sequelae of COVID-19 illness [11–14] and have documented the immune response to vaccination in immunocompromised patients [15] and the importance of vaccination (including booster vaccination) for reducing the risk of severe COVID-19 illness [16–18]. In the second instance, however, SARS-CoV-2 antispike protein (anti-S) serologic testing was not endorsed in the United States as having diagnostic or prognostic utility other than in assessing children and adults with potential multisystem inflammatory syndrome [19]. This perceived lack of utility for SARS-CoV-2 anti-S serologic testing is in contrast to use of serologic testing for surveillance of other vaccine-preventable pathogens [20], including evaluation of patients with immunocompromised status [21–25].

The COVID-19 Real World Data infrastructure (CRWDi) was funded by the National Cancer Institute (NCI) to address gaps in knowledge about the impact of COVID-19 on immunocompromised patients, either based on their underlying condition and/or because of immune-modulating therapies [26]. Established in May 2024 and containing 5.2 million unique patient records, the CRWDi data infrastructure constituted a de-identified, fully harmonized linkage of medical and pharmacy administrative claims data, vital status data, COVID-19 vaccination status data, and SARS-CoV-2 diagnostic and serologic laboratory data, with linkage also to deidentified patient records from the NCI Surveillance, Epidemiology, and End Results (SEER) network. Following an update in 2025, CRWDi spanned the COVID-19 pandemic for calendar years 2020 to 2024. Owing to the combined contribution of national data from the 2 largest national commercial laboratories, another commercial laboratory, and regional data from the largest health system in the greater New York metropolitan area, CRWDi thus constituted a robust repository of SARS-CoV-2 laboratory testing data that, in particular, provided a unique opportunity to address the utility of serologic testing in the assessment of patients made vulnerable through cancer, systemic autoimmune and rheumatic diseases (SARDs), and solid organ or stem cell transplantation. Using CRWDi, we report our strategies for successfully harmonizing these real-world laboratory data, with the goal of these strategies having general applicability for real-world study of the medical and public health utility of serologic testing.

## METHODS

### Source SARS-CoV-2 Laboratory Test Data

Two major commercial laboratories (Labcorp, Burlington, NC, USA; Quest Diagnostics, Secaucus, NJ, USA) contributed SARS-CoV-2 testing data, along with an integrated delivery network (Northwell Health, New Hyde Park, NY, USA) and a third commercial source for SARS-CoV-2 diagnostic testing only that was not disclosed to the authors. The inclusion dates for SARS-CoV-2 test data were March 1, 2020, to December 31, 2024. SARS-CoV-2 diagnostic testing was nucleic acid amplification testing (NAAT), reported qualitatively as negative or positive. Cycle threshold (Ct) values were not reported, and no antigen-based testing was included. SARS-CoV-2 serologic testing reported from the 3 identified laboratories included qualitative results for all test specimens and semiquantitative serologic test values on a subset of specimens.

### Normalization and Harmonization of SARS-CoV-2 Laboratory Data

The CRWDi data infrastructure was organized into relational tables; the laboratory relational tables included data fields for claim identification number, HealthVerity identification number (HVID), diagnostic codes entered on laboratory test requisitions, performing laboratory, date of test performance, test ordered name, test Logical Observation Identifiers Names and Codes (LOINC; Regenstrief Institute, Indianapolis, IN, USA), result name, result unit of measure, reference range, result, and result comment.

### Normalization of Test Name

The fundamental test categories were SARS-CoV-2 NAAT and SARS-CoV-2 serologic testing for total antigen, anti-S or antinucleocapsid (anti-N) antigens; “antispike” and “anti-RBD” were treated as “antispike.” Antibody class was either total antibody, immunoglobulin (Ig) G, IgM, or IgA. Although LOINC codes provided an initial scheme for identifying test type, substantial variation in test names remained. At the beginning of the pandemic, the SARS-CoV-2 serologic test was named “SARS-CoV-2.” However, as assays were further developed, tests were named with a more specific designation of whether the detected antibody was “anti-S” or “anti-N.” In similar fashion, in the early months of the pandemic, the serologic assays detected total immunoglobulin; specification of antibody class (IgG, IgM, and IgA) became available as different manufacturers stood up antibody class–specific assays and received regulatory approval. Supplementary Table 1 shows the multiplicity of test names requiring normalization (NAAT, n = 27; qualitative serology, n = 34; semiquantitative serology, n = 26).

### Parsing of Test Categories and Deduplication of Test Results

Following test name normalization, we applied logic for test result categorization and parsing (Table 1). However, all test categories had replicate entries for the same testing events. Specifically, for any given test result, the International Classification of Diseases, Version 10 (ICD-10), codes entered by the ordering physician on the test requisition were retained as unique data elements but populated separate rows for each test event. Hence, a test requisition with only 1 accompanying ICD-10 code was not replicated; a test requisition with 2 accompanying ICD-10 codes was reported in duplicate, and so on. Table 1 shows the breakdown of replicated SARS-CoV-2 test results in CRWDi for each test category, enabling us to determine the number of unique, deduplicated test results for each category. This coding logic is available to researchers to ensure that such replication is removed from future analytic work.

**Table 1.** Deduplication of SARS-CoV-2 Test Results

Rows per Result	Total Rows	NAAT Unique Results	Serology Qualitative Unique Results	Serology Semiquantitative Unique Results	Total Unique Results
1	6 497 860	3 581 269	2 260 204	656 387	6 497 860
2	3 143 262	1 127 334	397 696	46 601	1 571 631
3	626 595	75 601	113 939	19 325	208 865
4	564 620	53 523	71 613	16 019	141 155
5	436 360	11 983	59 054	16 235	87 272
6	395 694	5810	45 074	15 065	65 949
7	360 276	2544	35 507	13 417	51 468
8	323 544	2063	27 309	11 071	40 443
9	289 611	1163	21 449	9567	32 179
≥10	1 137 521	4141	60 353	30 800	95 294
Total	13 775 343	4 865 431	3 092 198	834 487	8 792 116

Data are presented for all test categories from the 4 source laboratories. The 7 cardinal data fields that described a unique test result were HealthVerity deidentified patient record number (HVID), administrative claim number, source laboratory, date of test, test name, test units, and test reference range (including for qualitative testing). However, replication of test results in CRWDi occurred due to multiple ICD-10 codes entered at the time of test ordering; each ICD-10 code generated a separate entry (row) for the test with which it was associated. Deduplication of test events (and hence rows in the relational table) required (a) identifying unique test events using the 7 cardinal data fields, (b) deduplicating multiple rows associated with single test events but that were created for each ICD-10 code on the laboratory test requisition, and (c) generating a deduplicated derivative table for SARS-CoV-2 laboratory test results in the CRWDi data infrastructure. This table shows the number of unique tests with singlicate rows in the source laboratory test table, those with duplicate entries, etc. Deduplication of this magnitude underscores the degree of systematic preprocessing of real-world data necessary to avoid inflating counts. Abbreviations: CRWDi, COVID-19 Real World Data infrastructure; ICD-10, International Classification of Disease, 10th Revision; NAAT, nucleic acid amplification testing; SARS-CoV-2, severe acute respiratory syndrome coronavirus 2.

### Normalization of Qualitative Test Results

Having parsed and deduplicated the test categories, there was substantive variability in how qualitative tests were reported. For both NAAT and serologic testing, the qualitative result should ostensibly be “negative” or “positive.” However, the alphanumeric characters in the results field were variable, with 59 different ways to report 1 of these 2 results (Supplementary Table 2). A complete inventory of all potential alphanumeric text sequences in the results field was compiled and verified with the source laboratories for their corresponding assignment to “negative” or “positive”; all qualitative results were recoded to the appropriate outcomes.

### Harmonization of Semiquantitative SARS-CoV-2 Serology Test Results

Table 2 then outlines our strategy for harmonizing semiquantitative serology test results. Starting with the parsing of test categories in Step 1, we selected only semiquantitative SARS-CoV-2 serologic tests. In Steps 2–4, for each source laboratory, we identified target antigen (“spike” or “nucleocapsid”), antibody class (“total,” “IgG,” “IgM,” or “IgA”), and manufacturer. For the 2 national commercial laboratories, this information was embedded in the results alphanumeric comment data field. Accordingly, this field was searched for manufacturer and assay names used during the study period. The natural language processing Arabic character sequences that were searched were “diasori” for Diasorin Inc. (Stillwater, MN, USA), “roche” for Roche Diagnostics (Indianapolis, IN, USA), and “siemens” for Siemens Healthineers (Erlangen, Germany). The serologic assays for these manufacturers are given in Supplementary Table 3. The CRWDi relational table for commercial laboratory semiquantitative test results was recoded to include a structured data field for each respective manufacturer for each test result. The test name, manufacturer, and date of service were then used to identify the reference range in use at that time, so as to identify those numerical test results below or at-and-above the reference values for positivity.

**Table 2.** Strategy for Harmonizing SARS-CoV-2 Testing Data

Logic for Test Result Harmonization	No. of Unique Test Results
Step 1. Eligibility for Serologic Data harmonization is determined by Test Type	
a. If Test = NAAT, then not Serology, and manufacturer assay is not determined	4 865 431
b. If Test = Serology qualitative, then manufacturer assay is not determined	3 092 198
c. If Test = Serology semiquantitative, then proceed to the next step	834 487
Step 2. For Quest, Manufacturer Assay is determined by Antigen, Immunoglobin, and Date of Service	Quest total = 193 773
a. If Antigen ≠ 'Spike,’ then manufacturer assay is not determined	27
b. If Immunoglobin = ‘Total immunoglobulins,’ then ‘Assay’ = ‘Roche Elecsys Anti-SARS-CoV-2 S’	40 480
c. If Immunoglobin = ‘IgG’ and date between ‘2021–02–15’ and ‘2021–10–31,’ then “Assay = ‘Siemens COV2G’”	65 769
d. If Immunoglobin = ‘IgG’ and date ≥ ‘2021–11–01,’ then “Assay = 'Siemens sCOVG’”	87 497
Step 3. For Labcorp, Manufacturer Assay is determined by Antigen and Test IDs	Labcorp total = 396 895
a. If Antigen ≠ 'Spike,’ then manufacturer assay is not determined	4
b. If Antigen = 'Spike’ and ‘Labcorp Test ID’ = ‘164090,’ then “Assay = ‘Roche Elecsys Anti-SARS-CoV-2 S’”	284 235
c. If Antigen = 'Spike’ and ‘Labcorp Test ID’ = ‘164055,’ then Assay = ‘DiaSorin (Trimeric S) IgG’	112 656
Step 4. For Northwell, Manufacturer Assay is determined by Antigen and Date of Service	Northwell total = 243 819
a. If Antigen = ‘Nucleocapsid,’ then ‘Roche Elecsys Anti-N’	33 434
b. If Antigen = ‘Total’ and date ≥ ‘2020–08–13,’ then ‘Roche Total Antigen’	59 299
c. If Antigen = ‘Total’ and date < ‘2020–08–13,’ then manufacturer assay is not determined	38 486
d. If Antigen = 'Spike,’ unit = ‘U/mL,’ and Reference range = ‘≤0.79,’ then Assay = ‘Roche Elecsys Anti-SARS-CoV-2 S’	112 600
Step 5. Binding Arbitrary Unit Conversion Factors are determined by Manufacturer Assay	

Manufacturer Assay	Conversion Factors	No. of Results
Roche Elecsys Anti-SARS-CoV-2 S	BAU = result/0.972	437 315
Siemens COV2G	BAU = (7.5145*[result^2^]) + (35.379*result) + 18.337	65 769
Siemens sCOVG	BAU = 45.078(result^0.7984^)	87 497
DiaSorin (Trimeric S) IgG	BAU = 2.6 * result	112 656
Roche Total Antigen	Not assigned	59 299
Roche Elecsys Anti-N	Not assigned	33 434
Manufacturer assay not determined	Not assigned	38 517

Data are given for all test categories from the source laboratories. The first step was to identify test category; if NAAT or qualitative SARS-CoV-2 serology (steps 1a, 1b, respectively), then the test manufacturer did not need to be known. If semiquantitative SARS-CoV-2 serologic test (step 1c), then use laboratory source to identify assay manufacturer. For Quest and Northwell (Steps 2 and 4, respectively), the calendar date of test performance was sufficient. For Labcorp (Step 3), a decode table was required. When the logic of step 1c yielded “null” for manufacturer test platform, harmonization of semiquantitative numerical results could not be performed. In Step 5, when the test category was a SARS-CoV-2 anti-S assay, the test result was associated with a known manufacturer test platform and the reference range for positivity was known, the published conversion factors for SARS-CoV-2 Anti-S testing [27] enabled harmonization of numerical values. Abbreviations: BAU, binding arbitrary units; NAAT, nucleic acid amplification testing; SARS-CoV-2, severe acute respiratory syndrome coronavirus 2.

For testing performed by Northwell Health, although semiquantitative serologic testing had been performed starting April 14, 2020, it was on multiple manufacturer platforms until early August 2020 [28], information that was not included in the CRWDi data infrastructure. The Roche platform was used exclusively after August 21, 2020. Northwell Health data reported herein are thus only for results obtained between August 21, 2020, and December 31, 2024.

Having identified the manufacturer, test platform, and reference range, semiquantitative numerical results that were below the manufacturer's reported threshold for positivity were recoded as “null,” and the below-threshold numerical test result was not carried forward. Only semiquantitative numerical test results at-or-above the manufacturer reference threshold, and therefore “positive,” were carried forward to Step 5. Published conversion factors were used to obtain harmonized BAU [27].

The source laboratories did not report a qualitative “indeterminate” designation for semiquantitative numerical test results just above the manufacturer reference threshold for positivity. The accompanying qualitative interpretation of semiquantitative SARS-CoV-2 serologic data was either “negative” or “positive.”

### Data Analysis

Using SQL programming language in the Databricks analytic environment of CRWDi (Databricks Inc., San Francisco, CA, USA), harmonized SARS-CoV-2 test data were joined with recode reference tables to specify the following: immunocompromised status (patients with cancer as identified by the SEER data registry; patients with SARDs as identified by ICD-10 coding who had received disease-relevant pharmaceutical therapy within the past year; patients who were not already included in the SEER-identified cancer cohort but who had undergone solid organ or stem cell transplantation based on ICD-10 codes; or a nonimmunocompromised general cohort [26]); test category; assay manufacturer; qualitative result (negative or positive, including the qualitative result accompanying a semiquantitative serologic numerical test result); serologic semiquantitative numerical test result when available; and calendar year and month the test was performed.

Results were visualized using R software (The R Project for Statistical Computing, R Foundation, Kaysville, UT, USA) and Excel (Microsoft, Redmond, WA, USA). For the purposes of charting ordinally ranked semiquantitative SARS-CoV-2 anti-S serologic numerical data on a semi-log plot and for performance of statistical analysis, negative (“null”) results were set to the numerical value of “1.” In the first instance, the value of “1” served as the lower limit for charting of harmonized BAU values on a semi-log y-axis. In the second instance, the value of “1” was used in the area-under-the-curve calculations for statistical analysis (see below).

As the scope of this study was harmonization of SARS-CoV-2 test results, we did not examine performance of multiple NAAT or serologic tests on the same human subject, nor the relationships of SARS-CoV-2 NAAT positivity or COVID-19 vaccination to SARS-CoV-2 serologic test positivity in individual human subjects.

### Statistical Analysis

To compare the distributions of semiquantitative SARS-CoV-2 anti-S serologic results across the 4 cohorts of patients with immunocompromising conditions and with the general cohort, ordinally rank-ordered harmonized BAU values were expressed as quantile functions evaluated at 201 evenly spaced x-axis cumulative fraction intervals ([0 to 1], in steps of 0.005). As noted in “Data Analysis,” y-axis BAU values below the manufacturer reference range for positivity were recoded to 1; all numerical values at or above the reference range for positivity were harmonized using the conversion factors shown in Table 2, Step 5.

For each cohort, we calculated the x-y area under the log_10_-transformed quantile curve on the cumulative fraction interval [0, 1] using the trapezoidal rule, thus obtaining the area under the curve (AUC). By a standard property of quantile functions, this integral approximates the mean log_10_(BAU) for that cohort [29], and its antilog gives the geometric mean BAU. Pairwise differences in AUC between each of the 4 immunocompromised cohorts and the general cohort were assessed by nonparametric bootstrap resampling with replacement (10 000 iterations). Two-sided *P* values were computed as the achieved significance level, and 95% CIs were determined using the 2.5th and 97.5th bootstrap percentiles [30, 31]. To account for the 10 pairwise comparisons between immunocompromised and general cohorts, we applied a Bonferroni correction, resulting in a family-wise significance threshold of *P* < .005.

### Data Availability

The deidentified aggregate data underlying this article will be shared on reasonable request to the corresponding author. The code used for the performance of this study will be made available upon agreement to collaborate with the first author on all publications that use such code to inform their own analyses and upon demonstration that the requestor is qualified and has documented evidence of training for human subjects protections.

### Patient Consent

The Institutional Review Board of Northwell Health approved the performance of this study under an exempt protocol and without requirement for patient consent.

## RESULTS

### SARS-CoV-2 Test Volumes

**Figure 1.**
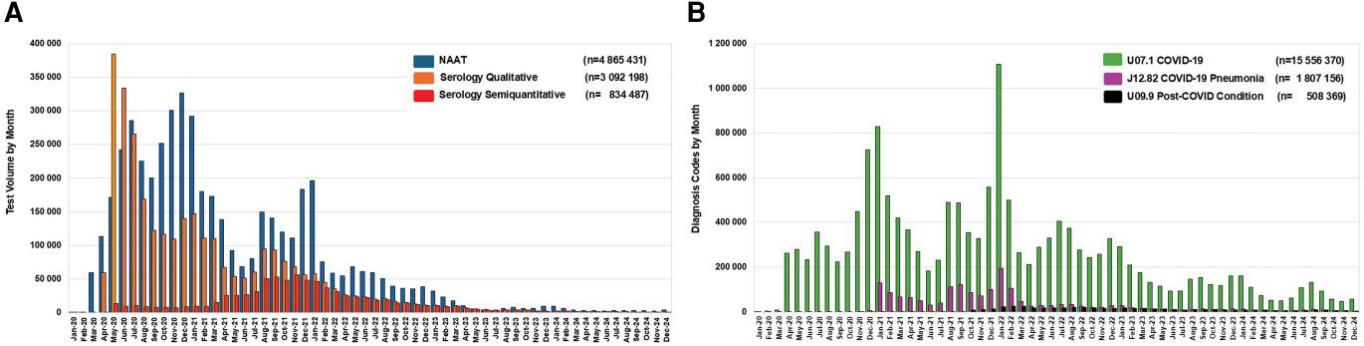
SARS-CoV-2 test volumes and COVID-19 ICD-10 coding volumes, 2020 to 2024. *A*, Test volumes by month and calendar year are shown for NAAT, qualitative serology, and semiquantitative serology; the legend gives total unique events for each test category. *B*, Unique ICD-10 coding events by month and calendar year are shown for the 5.2 million unique deidentified patients in the COVID-19 Real World Data infrastructure (CRWDi); the legend gives total unique coding events by ICD-10 code. Abbreviations: COVID-19, coronavirus disease 2019; ICD-10, International Classification of Diseases, 10th Revision; NAAT, nucleic acid amplification testing; SARS-CoV-2, severe acute respiratory syndrome coronavirus 2.

For the 5.2 million subjects in the CRWDi data resource, there were a total of 8 792 116 unique SARS-CoV-2 testing events (Table 3), shown by month and year in Figure 1*A*. The preponderance of testing events was in calendar years 2020–2022. For SARS-CoV-2 serologic testing, qualitative predominated in 2020 to mid-2021. From November 2021 onwards, semiquantitative testing usually accompanied qualitative test reporting.

**Table 3.** SARS-CoV-2 Test Volumes by Source Laboratory, Test Category, and Test Subcategory

Test Category	Northwell	Quest	Labcorp	Other	Total
NAAT	416 975	1 717 297	1 786 820	944 339	4 865 431
Serology qualitative	243 777	1 039 579	1 808 842		3 092 198
Total IgG	97 538	840 460			937 998
Total IgM		54 359			54 359
Isotype not provided		303			303
Spike Total	112 833		260 024		372 857
Spike IgG	3	40	1 046 443		1 046 486
Spike IgM		55 353	287 770		343 123
Spike IgA			75 991		75 991
Nucleocapsid Total	33 403	397	138 614		172 414
Nucleocapsid IgG		88 667			88 667
Serology semiquantitative	243 819	193 773	396 895		834 487
Total IgG	97 785	16			97 801
Spike Total		40 480	284 235		324 715
Spike IgG	112 600	153 266	112 656		378 522
Nucleocapsid Total			4		4
Nucleocapsid IgG	33 434	4			33 438
Total IgM		7			7
Total	904 571	2 950 649	3 992 557	944 339	8 792 116

Following the deduplication shown in Table 2, the unique test events in the CRWDi data infrastructure are shown. Northwell, Quest, and Labcorp reported all 3 categories of test results; 1 other laboratory source reported only qualitative SARS-CoV-2 NAAT testing and was not included in the methodology for harmonizing SARS-CoV-2 serologic testing data. Abbreviations: CRWDi, COVID-19 Real World Data infrastructure; NAAT, nucleic acid amplification testing; SARS-CoV-2, severe acute respiratory syndrome coronavirus 2.

### COVID-19 ICD-10 Coding Events

Supplementary Table 4 shows the ICD-10 codes relevant to COVID-19 illness that accompanied the SARS-CoV-2 test requisitions. The predominant ICD-10 codes were the following: asymptomatic screening (n = 584 901) including screening for COVID-19 (Z11.52, n = 47 386); contact (n = 1 624 364) including exposure to COVID-19 (Z20.822, n = 483 708); and symptoms or disease condition (n = 500 454) including COVID-19 illness (U07.1, n = 114 195). These potentially relevant ICD-10 codes accompanying SARS-CoV-2 test orders (n = 2 709 719) are far fewer in number than the total testing events (n = 8 792 116). It is also notable that the ICD-10 code for “post-COVID condition, unspecified,” U09.9, was not present in the requisition orders for SARS-CoV-2 NAAT or serologic testing.

Figure 1*B* shows the total ICD-10 coding in the CRWDi database, as obtained both from administrative claims data and from laboratory test requisitions, by month and year. Three COVID-19-specific codes are shown: U07.1 (COVID-19 illness, n = 15 556 370), J12.82 (COVID-19 pneumonia, n = 1 807 156), and U09.9 (post-COVID condition, n = 508 369). The vast preponderance of the 17 477 721 total ICD-10 codes for COVID-19 (U07.1, J12.82, U09.9) were thus from administrative claims data.

### Semiquantitative SARS-CoV-2 Anti-S Serologic Testing

**Figure 2.**
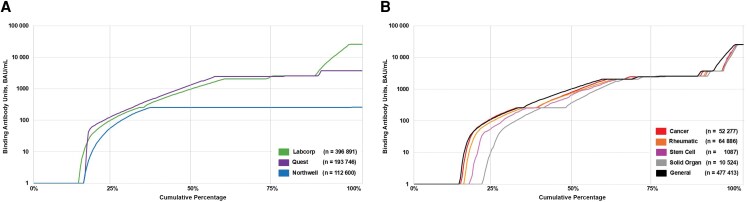
Cumulative semi-log ordinal plots of unique testing events for semiquantitative SARS-CoV-2 anti-S serology by source laboratory (*A*) and comparisons of the distributions of ordinally rank-ordered, harmonized BAU values from semiquantitative SARS-CoV-2 anti-S serologic tests across 4 cohorts of patients with immunocompromising conditions and the general cohort (*B*). The x-axis shows the cumulative percentage of testing events. The y-axis shows the harmonized BAU, per the logic given in Table 2. The legends give the total unique testing events for each curve. For both (A) and (B), the value of 1 on the semi-log y-axis denotes a “negative” test result. For (B), on a pairwise comparison basis, each of the curves is significantly different than each of the other curves (*P* < .001; see “Statistical Analysis” in the “Methods” section). Abbreviations: BAU, binding arbitrary units; SARS-CoV-2, severe acute respiratory syndrome coronavirus 2.

Harmonization of semiquantitative SARS-CoV-2 anti-S serologic testing enabled visualization of semiquantitative test results using cumulative frequency semi-logarithmic plots. The purpose of this visualization was to provide immediate appreciation of the percentage of test results that were negative, submaximal (falling in the dynamic range of the semiquantitative assay), and maximal (above the dynamic range). Figure 2*A* shows the cumulative percentage of results from the 3 laboratories contributing serologic data. The dynamic range was 0–250 BAU for Northwell Health test results, for testing performed entirely on the Roche platform, and without autodilution of specimens that resulted >250 BAU. This chart documents that Quest routinely performed 1:10 autodilution, with a harmonized dynamic range of 0–2500 BAU; the slightly variable plateaus represent different conversion factors for the different manufacturer platforms. In turn, Labcorp performed both 1:10 and 1:100 autodilutions, as documented by the plateaus both in the 2500 and 25 000 BAU ranges. Despite these differing autodilution practices between the 3 source laboratories, the data show that the percentages of test results that were negative were comparable for the 3 laboratories. For Quest and Labcorp, anti-S antibody curves were similar up to the 1:10 dilution plateaus of ∼2500 BAU. Lastly, for those Labcorp test specimens that were diluted 1:100, only a limited percentage resulted at the maximum of 25 000 BAU.

We next examined semiquantitative serologic anti-S test data based on indexed immunocompromised status (Figure 2*B*). Most serologic tests were performed on a comparison general population: 13% exhibited harmonized BAU test results below the reference threshold for positive; from 13% to 57%, the test results were in a submaximal range of 1–2500 BAU; and above 57%, test results were maximal at ≥2500 BAU. For the 4 immunocompromised cohorts, 14% of specimens from patients with cancer had negative test results, compared with 15% for patients with SARDs, 17% for patients who had undergone stem cell transplant, and 21% for patients who had undergone solid organ transplant patients. The cumulative distributions in the submaximal dynamic range lagged similarly for the 2 transplant cohorts. Once test results plateaued in the ≥2500 BAU range, the curves were largely similar, although there was separation of the general population cohort in the rise from 2500 to ≥25 000 BAU.

## DISCUSSION

Harmonization of laboratory testing is a foundational principle for the practice of laboratory medicine [32]. However, while analytical standardization of results from clinical laboratories can be achieved through metrological traceability of results to certified reference materials or reference measurement procedures [33], specifics regarding the naming of tests for ordering and reporting are the prerogative of individual laboratories. In this instance, laboratories were implementing testing in response to a pandemic, with new tests and platforms being approved at a rapid rate, for an unknown level of demand for testing capacity. The fact that 4 source laboratories generated 87 different test names and 59 different ways to report “positive” or “negative” provides an important learning opportunity relevant to preparedness for future pandemics, when testing platforms and regulatory approvals may be changing rapidly. Beyond that, we consider these strategies to be of general relevance to harmonization of real-world laboratory data. Our recommendation is that, when creating a harmonized data infrastructure involving qualitative, semiquantitative, and/or quantitative laboratory test information, careful attention should be given to test names, units, reference ranges, and manufacturers, requiring these fields to be part of the data intake. The actual contents of the result fields then need to be validated for alphanumerical text and numerical value interpretability. This information will then facilitate both normalization of the structured data through the process of recoding and harmonization of the actual results, taking advantage of conversion factors and/or standard reference information that may be available.

While the LOINC coding system aims to standardize both the test name and the specifics of the assay platform [34], the above pluralities show that there is insufficient structured content in LOINC to permit unsupervised harmonization of test names and even qualitative test results [35]. Semiquantitative serological testing is a particular challenge as, until a serological assay is harmonized to an international standard by the manufacturer, results are given in BAU specific to each manufacturer. Although World Health Organization (WHO) standards are in place for reporting of serologic test results for other vaccine-preventable viral pathogens [36–38], standardization is not yet in place for SARS-CoV-2 serological testing [39, 40]. Beyond that, the August 29, 2024, recommendations of the Centers for Disease Control and Prevention (CDC; [18]) are essentially unchanged from the 2020 recommendations: SARS-CoV-2 serologic testing does not diagnose current infection and is primarily used for public health surveillance and epidemiologic purposes. Per the CDC recommendations, the presence of SARS-CoV-2 antibodies may aid in fulfilling the case definition for multisystem inflammatory syndrome in children (MIS-C) and adults (MIS-A) and may help in distinguishing an immune response from natural infection vs vaccination. US guidelines remain silent on the interpretation of semiquantitative SARS-CoV-2 serologic testing, especially its role in assessing patients with immunocompromising conditions. Barriers to establishing such utility include the differing assay cutoffs for negative and positive results, the impact of SARS-CoV-2 variants on host immune response, the absence of established correlates of protection, and lack of clarity on the role of T cells in preventing severe disease [25]. The methodology described herein for harmonization of SARS-CoV-2 laboratory tests may make such research possible.

Several observations can be made from the data presented herein. First, the increase in qualitative serologic testing in May, June, and July 2020 is likely a reflection of the serosurveillance studies performed in the early months of the pandemic [41], including health care workforce studies [28]. The substantial decrease in calendar years 2023 and 2024 of both laboratory-based NAAT and serology testing likely reflects utilization of over-the-counter antigen testing for home-based COVID-19 diagnosis and the lack of indication for testing SARS-CoV-2 antibody levels in clinical practice. This is despite COVID-19 being involved in 20 677 (0.7%) of the 3.1 million total deaths in the United States in 2025 [42] and of continued relevance in examining excess mortality in the United States in the post-COVID era [43].

Second, the preponderance of COVID-19 illness–related ICD-10 codes being provided by administrative claims data underscores the value of harmonizing SARS-CoV-2 laboratory testing with administrative data to understand test utilization. Third, while the performance or not of autodilution of serologic specimens by the source laboratories may have differed (Figure 2*A*), the overall similarities of the semi-log ordinal curves support the premise that the source laboratories are testing populations of similar composition. This provides justification for merging of the respective data sets for further studies. Fourth, Figure 2*B* documents that specimens from patients with stem cell transplantation and, especially, solid organ transplantation exhibit a higher percentage of negative results and a lag in the number of specimens that reach maximal values over the dynamic range of the semiquantitative assay when compared with the general population and with specimens from patients with cancer or SARDs. Although we speculate that recipients of solid organ transplants will be on immunosuppressive regimes in time frames proximate to the performance of SARS-CoV-2 serologic testing, it will be for future studies to determine the relationships of semiquantitative serologic test results with patient status.

The limitations of this study are as follows. First, this is a methodological report of harmonization of unique test results and is not an actual study of the clinical utility of the testing. This includes the fact that vaccination status and history of SARS-CoV-2 infection were not part of our analysis. Second, variable autodilution practices for anti-S serological results (Figure 2A) may limit interpretation of maximal test results. However, we consider that patients with negative or submaximal antibody levels are of great interest for future studies, on the basis of an absent or lower humoral response. Third, the indexing of patients by condition (Figure 2B) does not consider the impact of the immunomodulating therapy that these patients may have received. It will be of importance to include the immunosuppressive regimes being received by patients as a covariable in future studies using serologic data. Last is acknowledgement that multiple serological assays were used in creation of the CRWDi data infrastructure. Studies of the utility of serological assays in identifying correlates of protection have benefited from standardization of assays, noting that use of multiple testing platforms for vaccine-preventable pathogens is common [36–38]. However, it is precisely because the COVID-19 pandemic forced the creation by multiple manufacturers and clinical laboratories of as much capacity as was possible while evolving through the technical requirements for testing a novel pathogen, that the real world of SARS-CoV-2 serologic testing exhibited such profligate variety. Our hope is that the current work will be of specific value for future events in which multiple diagnostic assays are in play over the course of a rapidly evolving public health emergency.

## CONCLUSIONS

This report provides a generalizable methodology for harmonizing qualitative and semiquantitative laboratory testing data for a vaccine-preventable pathogen, in this case SARS-CoV-2. Both diagnostic and serological testing data from multiple laboratory sources can be successfully harmonized with the broad geography of real-world administrative claims and vital status data. For CRWDi, this also included harmonization with cancer diagnostic, therapeutic, and longitudinal outcomes data from the SEER registry system, representing a particular enhancement of this resource. This report also demonstrates the specific need for harmonization of real-world laboratory testing data when testing was performed during the rapidly evolving time frame of a pandemic.

## Supplementary Material

ofag454_Supplementary_Data

## Supplementary Data

Supplementary materials are available at *Open Forum Infectious Diseases* online. Consisting of data provided by the authors to benefit the reader, the posted materials are not copyedited and are the sole responsibility of the authors, so questions or comments should be addressed to the corresponding author.
